# Soil-Driven Coupling of Plant Community Functional Traits and Diversity in Desert–Oasis Transition Zone

**DOI:** 10.3390/plants14131997

**Published:** 2025-06-30

**Authors:** Zhuopeng Fan, Tingting Xie, Lishan Shan, Hongyong Wang, Jing Ma, Yuanzhi Yue, Meng Yuan, Quangang Li, Cai He, Yonghua Zhao

**Affiliations:** 1 College of Forestry, Gansu Agricultural University, Lanzhou 730070, China; fanzp5532@163.com (Z.F.); hongyongwang00@gmail.com (H.W.); majinggabriel@gmail.com (J.M.); yueyuanzhi123@163.com (Y.Y.); yuanmeng1882710@163.com (M.Y.); liquangang2024@126.com (Q.L.); 2 Wuwei Academy of Forestry, Wuwei, 733000, China; hcyldfcl@163.com; 3 School of Land Engineering, Chang′an University, Xi’an 710054, China; yonghuaz@chd.edu.cn

**Keywords:** plant community, functional trait, species diversity, functional diversity, adaptation strategy, desert–oasis transition zone

## Abstract

Understanding the relationships between diversity and functional traits in plant communities is essential for elucidating ecosystem functions, forecasting community succession, and informing ecological restoration efforts in arid regions. Although the current research on plant functional traits and diversity has improved our ability to predict ecological functions, there are still many problems, such as how environmental changes affect the relationship between species diversity and plant functional traits, and how these interactions affect plant community functions. We examined the relationships among leaf and fine root functional traits, species diversity, and functional diversity at the community level, along with their environmental interpretations, in a plant community within the desert–oasis transition zone of the Hexi Corridor, where habitats are undergoing significant small-scale changes. During dune succession, plant community composition and diversity exhibited significant variation. Plants are adapted to environmental changes through synergistic combinations of above-ground and below-ground traits. Specifically, plants in fixed dunes adopted a “slow investment” strategy, while those in semi-fixed and mobile dunes employed a “fast investment” approach to resource acquisition. A strong coupling was observed between plant community functional traits and species diversity. Soil phosphorus content and compactness emerged as primary factors influencing differences in plant community functional traits and composition. These soil factors indirectly regulated fine root functional traits and diversity by affecting species diversity, thereby driving community succession. Our study elucidates the “soil—diversity—community functional trait” linkage mechanisms in the successional process of desert plants. This research provides scientific support for the restoring and reconstruction of degraded ecosystems in arid zones.

## 1. Introduction

Climate change and human activities have significantly altered the habitats of plant communities, impacting their composition, successional dynamics, and soil nutrient profiles [1,2]. As plants adapt to these environmental changes through variations in functional traits and inter-trait relationships [3], their associated functional diversity also shifts, leading to adjustments in the coupling between functional traits and diversity within plant communities. Extensive evidence indicates that habitat degradation often results in biodiversity loss and diminished ecosystem functions [4,5]. Analyzing the coupling between plant community functional traits and diversity is crucial. It helps us understand how plants adapt to changing habitats. It also reveals the mechanisms that drive community responses. These can guide the restoration of degraded ecosystems.

Ecosystems typically undergo structural and functional variation in response to environmental changes; this process can be measured by the formation of plant diversity over long periods of evolution [6]. However, plant diversity is a multifaceted concept. Owing to the inherent variability in species morphology, physiology, and traits, research has shifted from focusing solely on species count—such as species richness and distributional evenness [7]—to emphasizing variations in the functional diversity within communities. In addition, in response to environmental changes, plant community functional traits comprehensively reflect species’ abilities to acquire resources and adapt their ecological strategies [8,9]. Their diverse expressions across various environments suggest that environmental factors may influence plant community structure and functions by shaping these traits, which leads to their widespread use in functional diversity studies. Therefore, analyzing key traits in leaves and fine roots related to resource acquisition and conservation can aid in predicting and assessing the resilience of plant communities to disturbances.

The turnover of plant communities along abiotic gradients, encompassing both species and functional compositions, has become a central focus in ecological research [10,11]. To understand how environmental changes influence the assembly and succession of plant communities, functional diversity is often examined by the mass–ratio hypothesis and the diversity hypothesis. According to the mass–ratio hypothesis, the functional performance of an ecosystem is largely governed by the traits of dominant species [12,13]. Building on this framework, Garnier et al. [14] proposed a community-weighted mean trait (CWM) based on the biomass ratio hypothesis in order to test the mass–ratio hypothesis, which integrates individual plant functional traits with species abundance to assess functional diversity by mean community weights. Functional diversity reflects the degree of variation in functional traits of a plant community [15], and the diversity hypothesis [16] suggests that greater variation in functional traits within a community reduces niche overlap among species, facilitating coexistence and enhancing ecosystem functions. Previous studies showed that variations in CWM and functional diversity indices are good indicators to quantify ecosystem response to environmental changes and will show different patterns along environmental resource gradients such as light, temperature, moisture, elevation, and soil [17].

Variations in the combinations and trade-offs of multiple traits within plant communities reflect the adaptive strategies plants employ to respond to environmental pressures [18,19]. Previous studies, constrained by challenges in sampling and measurement techniques, have predominantly focused on analyzing species’ survival strategies and coexistence mechanisms based on above-ground traits, leaving the role of root traits in community ecology relatively unexplored. However, focusing solely on above-ground traits is insufficient to fully explain species coexistence mechanisms, as plants experience distinct selection pressures above-ground and below-ground during growth [20], and root traits are more influenced by soil texture, moisture, and nutrient availability. In addition, in the unstable desert environment, plants enhance their survival and expansion capabilities through asexual reproduction structures such as stolons or rhizomes [21,22]. Plants also utilize well-developed storage organs (tubers, bulbs, thick rhizomes, etc) to store water and nutrients during adverse conditions. When water and other conditions improve during the rainy season, they rapidly complete their growth and reproduction cycles, thereby restoring and maintaining the community structure through this strategy [23].

Recent advancements in measurement methods and the conceptualization of root orders [24] have led to a broader recognition among scholars that the root system comprises a complex of individuals with varying traits and functions. Significant trait differences among root sequences can help us better understand plant resource acquisition and adaptation strategies. Moreover, leaves and fine roots are the most important plant organs above- and below-ground [25,26]. Their nutrient utilization strategies are closely connected. This connection plays a key role in species distribution, community structure, and ecosystem functions.

In extreme habitats, leaves and fine roots exhibit complex relationships characterized by both trade-offs and synergistic changes [27], or transitions from trade-offs to synergies along environmental gradients [26]. For instance, under reduced ambient temperature and soil moisture, plants mitigate stress by enhancing nutrient content in both above- and below-ground organs [27]. Above-ground organs show decreased specific leaf area and increased leaf dry matter content [28]. In contrast, below-ground organs display increased root diameter and lateral root length, accompanied by decreased root branching intensity [29]. These patterns align with the concepts of leaf economic spectrum (LES) [30] and root economic spectrum (RES) [31], which emphasize the trade-offs between leaf and root for resource acquisition and conservation. Recent studies have advanced our understanding of plant functional trait economics. In the Himalayas, *Parthenium hysterophorus* L. expands its functional ecological niche with increasing altitude by adopting resource utilization strategies that enhance SLA, leaf thickness, and leaf nitrogen and phosphorus content [32]. In the Mediterranean region, along resource gradients, there is a strong synergy between above- and below-ground plant parts, resulting in an “economic spectrum of plant communities” driven by nutrients and water [33]. These studies underscore the importance of applying trait-based economic profiling methods to assess plant community variation to environmental changes.

In addition, the functional similarity hypothesis posits coordinated variation between root and leaf traits, as leaf growth depends on the transport of soil resources from the root system, and root growth depends on the distribution of leaf photosynthetic products [34,35]. However, recent studies have found that coordination between leaf and root traits is low, and their relationships are confusing [36]. Some scholars also contradicted the idea of a high degree of integration of above-ground and below-ground traits in plants. For example, Hobbie et al. [37] found no correspondence between leaf and root chemical traits, while Liu et al. [38], Li et al. [39], and Valverde-Barrantes et al. [40] reported positive, negative, and uncorrelated relationships between SLA and specific root length, respectively. Therefore, further research is needed to validate the correlations between analogous paired functional traits above- and below-ground in plants. Despite extensive global research on plant functional traits, studies focusing on specific habitats, such as the desert–oasis transition zone, remain limited.

In the desert–oasis transition zone of the Hexi Corridor, understanding plant community responses to environmental changes is especially critical, as intensified human land use and climate shifts have dramatically altered this landscape. Over the past three decades, the oasis area in the Hexi Corridor has increased by approximately 40% [41], substantially modifying the original natural environment. Concurrently, unregulated development has inflicted severe ecological damage, exacerbating desertification threats throughout the corridor [42]. As desertification increases (from fixed dunes to shifting dunes), vegetation becomes sparse and thin. The soil environment deteriorates significantly. The overall ecosystem becomes more barren and less diverse [43]. Many believe that moisture differences drive the variations in vegetation distribution and composition. The desert–oasis transition zone acts as an ecological buffer between mobile desert and cultivated oasis ecosystems [44], a natural barrier to maintain the stability of oasis agricultural production and habitat security [45]. Desert–oasis transition zones form highly heterogeneous habitats at smaller scales, and their plant communities play an important role in ensuring the ecological security of oases [46]. Although previous work has detailed leaf–root trait coordination in individual species [47], and reported extensively on the formation of the “fertile island effect” mechanism and its impact on desert ecosystems [48,49], research on the use of community-based methods and functional diversity indices based on multiple degrees and traits to explain the correlation between community succession and soil factors is still scarce. Therefore, priority should be given to assessing how the functional structure of plant communities in this transition zone responds to environmental change, to inform effective desertification control strategies.

This study aimed to assess variations in plant community functional traits and diversity across a regional habitat gradient in the desert–oasis transition zone of the central Hexi Corridor, and to infer the potential consequences of climate change and ecological degradation in a fragile, sensitive, and vulnerable area. We studied plant communities in the transition zone of a desert–oasis in the central Hexi Corridor, and we assumed the environmental gradient acts as a strong filter. It affects both the species composition and functional structure of these communities. We investigated the key traits related to the strategies of plants in coping with stress, and explored the effects of localized habitat changes on the functional traits and plant diversity of desert plant communities during different stages of fixation of dunes by analyzing their relationships and environmental interpretations. We expect to find that: (i) plant community composition would differ in response to habitat changes, and plant communities form different trait combinations when responding to abiotic stress. In mobile sand dunes, traits related to rapid resource acquisition are predominant, while in fixed sand dunes, conservative traits are dominant; (ii) A strong coupling would exist between functional traits and plant diversity under harsher soil conditions, with soil moisture differences acting as key drivers of variations in functional traits and community composition across different stages of dune fixation. The findings of this study will provide a theoretical foundation for desertification control and ecological restoration efforts.

## 2. Materials and Methods

### 2.1. Study Site

The study area is situated near the Linze Inland River Basin Comprehensive Research Station of the Northwest Institute of Eco-Environment and Resources, Chinese Academy of Sciences, at geographical coordinates 39.22°–39.24° N latitude and 100.08°–100.14° E longitude. It lies at the confluence of the Badain Jaran Desert and the Linze Oasis within the Heihe River Basin of the Hexi Corridor, characterized by a typical temperate continental arid desert climate. The region experiences an annual average temperature of 7.6 °C, annual precipitation of 117 mm, and evaporation rates of 2390 mm, resulting in a drying index of 20. The predominant soil type is gray-brown desert soil, which is coarse-textured, low in nutrients, and generally poor in quality.

### 2.2. Sample Collection

To collect samples during the plant growing season, we selected three habitat types unaffected by grazing: fixed dune (FD), semi-fixed dune (SFD), and mobile dune (MD), with vegetation coverages of ≥30%, 10–30%, and <10%, respectively [50]. In this study, all species within each quadrat were included, and three plots (20 m × 20 m) were established for each habitat type. To minimize the impact of environmental temperature on plant traits, the plots were spaced no more than 10 km apart. Within each sample plot, the “plum blossom” sampling method was employed at the four corners and center to select five 5 m × 5 m quadrats, totaling 45 quadrats across 9 sample plots. We assessed plant community characteristics by recording total coverage, species composition, and individual species coverages, documenting all plant species and their quantities within the quadrats. Based on the sample quadrats data, the sample plots were summarized as a community. Following Cheng et al. [51], species with relative abundances between 1% and 5% were classified as common, while those with relative abundances exceeding 5% were considered dominant. Vegetation characteristics of dunes at different fixation stages were summarized in Table 1. In each plot, 5 to 10 mature, healthy plants free from pests and diseases were randomly selected for analysis. For shrub species, fine roots were collected using the fine root tracing method: first, the main root was exposed with a spade, then fine roots connected to it were excavated downward or around, removing sediment near the root tips carefully to prevent damage, and collecting complete fine roots with more than three orders by cutting with scissors, followed by thorough cleaning. For herbaceous species, fine roots were collected within 10 to 20 cm of the stem base by digging a 0–30 cm deep soil block with a spade, extracting the entire plant including roots, and cleaning soil and impurities meticulously to preserve root integrity. Leaf and root samples from at least three plants per species were collected in each plot. After cleaning, fine roots and leaves were placed in labeled self-sealing bags, stored in coolers with ice packs to maintain activity, and transported to the laboratory for refrigeration until analysis of morphological and chemical properties.

### 2.3. Measurement of Plant Functional Traits and Soil Indicators

#### 2.3.1. Measurement of Leaf Functional Traits

Dust and other impurities were removed from the leaves, which were then scanned with a high-precision scanner to capture their morphological features. Leaf area (LA, cm^2^) was measured using ImageJ 1.53k software, and leaf volume (LV, cm^3^) was determined by the drainage method. Subsequently, the leaves were sealed in envelope bags, then dried at 75 °C for 48 h until a constant weight was reached, and finally weighed to record the leaf dry mass. Specific leaf area (SLA, cm^2^/g) was computed as the ratio of leaf area to leaf dry mass, and leaf tissue density (LTD, g/cm^3^) as the ratio of leaf dry mass to leaf volume. The dried leaves were then crushed and sieved through a 100-mesh sieve to prepare test samples. Total carbon content of leaves (LCC, mg/g) was determined using the heating method with potassium dichromate oxidation. Total nitrogen content (LNC, mg/g) was determined using the Kjeldahl method. Total phosphorus content (LPC, mg/g) was determined using the Mo-Sb colorimetric method.

#### 2.3.2. Measurement of Fine Root Functional Traits

Fine roots collected from intact plants were initially washed with deionized water to remove impurities (e.g., adhering sediment), and excess moisture was removed using absorbent paper. The roots were then classified into different orders following the method of Pregitzer et al. [24]: the most distal root tip was designated a primary root, the intersection of two primary roots as a secondary root, and the intersection of two secondary roots as a tertiary root. Root images were scanned and analyzed using Win-RhIZO 2008a software to determine fine root lengths, and fine root volume (RV, cm^3^) was subsequently measured by the drainage method. Finally, the fine roots were sealed in envelope bags, dried at 75 °C for 48 h until a constant weight was attained, and then weighed to record the dry mass. Specific root length (SRL, cm/g) was calculated as the ratio of total fine root length to fine root dry mass, while fine root tissue density (RTD, g/cm^3^) was determined as the ratio of fine root dry mass to fine root volume. Root carbon content (RCC, mg/g), root nitrogen content (RNC, mg/g), and root phosphorus content (RPC, mg/g) were determined using the same methods as those applied for leaves. Three replicates were established for both leaves and roots, and the experimental measurement methods of Chen et al. were referenced [47].

### 2.4. Measurement of Soil Indicators

Within each sample plot, after removing the surface layer of withered material and humus, soil samples were collected using a 3 cm diameter soil auger at the four corners and center to a depth of 0–60 cm. The collected soil was homogenized using the tetrad method, impurities were removed (e.g., roots, stones, and plant or animal residues), then the soil was sieved through a 100-mesh sieve and stored in labeled self-sealing bags for subsequent analysis of soil indices. The soil samples were weighed to determine their fresh weight, oven-dried at 105 °C until a constant weight was reached, and then reweighed to obtain the dry weight. Soil organic carbon content (SCC, mg/g), soil nitrogen content (SNC, mg/g), and soil phosphorus content (SPC, mg/g) were determined using methods analogous to those used for plant samples. Soil moisture content (SMC, %) was measured using the drying and weighing method (differential subtraction method), and soil compactness (SC, Pa) was assessed with a soil compactness meter (Crimik P40 II). The average value from three replicates per soil sample was used for analysis, and the data for each sample plot represent the mean of five sample plots. The basic situations of soil at different stages of sand dune fixation are shown in Table 1.

### 2.5. Species Diversity Calculations

In this study, four commonly used diversity indices were employed to assess the species diversity of plant communities at different stages of sand dune fixation: Margalef’s index (*M*) [52], Shannon–Wiener’s index (*H*) [53], Simpson’s index (*D*) [54], and Pielou’s evenness index (*J*) [55]. These indices were calculated using the following formulas:

(1) M=(S−1)/lnN

(2) $$H=-\sum \left(n_{i}/N\right)\times ln\left(n_{i}/N\right)$$

(3) $$D=1-\sum {\left(n_{i}/N\right)}^{2}$$

(4) J=H/lnS

In the formulas, *S* represents the number of species in the plant community within the sample, *N* is the total number of plant individuals surveyed, and *n_i_* denotes the number of individuals of the *i*-th species.

### 2.6. Calculation of Community Mean Trait Values

In this study, we calculated the community-weighted mean (CWM) trait values for each of the nine communities (n = 9) by assigning plant species (s = 16) based on their relative abundance. Each sample plot served as the basic unit for this calculation [12]. These indices were calculated using the following formula:

(5) $$CWM=\sum_{i=1}^{n}P_{ij}{\times trait}_{ij}$$

In the formula, CWM represents the community-weighted mean, calculated as the average trait value of the community; *trait_ij_* denotes the trait value of species *i* within community *j*; *P_ij_* indicates the relative abundance of species *i* in community *j*; and *n* signifies the total number of species in the community.

### 2.7. Functional Diversity Calculations

In this study, 10 functional traits were selected to assess the functional diversity of plant communities at different stages of dune fixation. These traits were: specific leaf area, leaf tissue density, leaf carbon content, leaf nitrogen content, leaf phosphorus content, specific root length, fine root tissue density, fine root total carbon content, fine root total nitrogen content, and fine root total phosphorus content. To characterize functional diversity, four indices were employed: functional richness (FRic), functional evenness (FEve), functional divergence (FDiv), and Rao’s quadratic entropy (RaoQ) [56,57]. Calculations were performed using the “fundiversity” package in R version 4.4.2.

### 2.8. Statistical Analyses

Data preprocessing was conducted using Microsoft Excel 2019. Species diversity indices and community-weighted mean (CWM) trait values were calculated accordingly. In R4.4.2, the functional diversity index was calculated, and Bio-Env analysis was performed using the “vegan” package. The Bio-Env analysis was performed by calculating Spearman’s rank correlation between the Bray–Curtis dissimilarity matrix of species data and the Euclidean distance matrix of environmental variables. Pearson correlation analysis was employed to examine the relationships between plant functional traits and community-level plant diversity. Multiple stepwise regression analysis was utilized to further assess the impact of plant community functional traits on plant diversity. Redundancy analysis (RDA) was conducted in Canoco 5.0, employing a Monte Carlo permutation test to evaluate the influence of soil factors on functional traits and their contributions. Finally, structural equation modeling was applied to assess the effects of soil factors, leaf functional traits, fine root functional traits, and species diversity on functional diversity. Partial least squares structural equation models were constructed using the “lavaan” package in R (version 4.4.2).

## 3. Results

### 3.1. Vegetation Characteristics and Plant Diversity at Different Stages of Fixation in Sand Dunes

All species and information from the vegetation survey are shown in Table 2. A total of 15 plant species were recorded in the desert–oasis transition zone during this survey, representing 7 families and 15 genera. The community composition included 5 shrub species, 5 perennial herb species, and 5 annual herb species. Vegetation characteristics and species relative abundance at different stages of stabilization for each sand dune are shown in Table 3. The fixed dune habitats have the highest number of species (14), followed by semi-fixed dune habitats with 5 species, and mobile dune habitats have the lowest number of species (3). The fixed dune habitats exhibited the highest species diversity among the three habitat types, with a predominance of shrub species. Notably, *Nitraria sphaerocarpa* was present across all three habitats, indicating its broad ecological tolerance.

**Figure 1:**
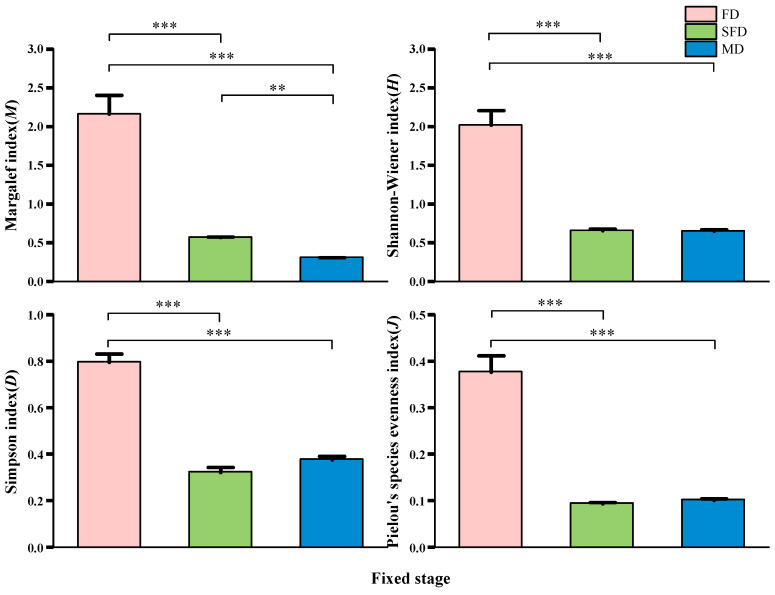
Species diversity of plant communities at different stages of fixation in sand dunes. Error bars indicate the standard error of the mean. Asterisks indicate significant differences (** *p* < 0.01 and *** *p* < 0.001). FD: Fixed dune; SFD: Semi-fixed dune; MD: Mobile dune.

Comparison of species diversity indices across different dune fixation stages revealed that the fixed dunes of natural communities in the desert–oasis transition zone exhibited significantly higher species diversity than semi-fixed and mobile dunes (Figure 1). In summary, the Margalef, Shannon–Wiener, Simpson, and Pielou evenness indices were all the highest in the fixed dune habitats.

Comparison of species diversity indices across different dune fixation stages revealed that the four functional diversity indices showed significant differences (*p* < 0.05) (Table 4). The FRic indices of mobile sand dunes were significantly higher than those of fixed and semi-fixed sand dunes. The FEve, FDis, and RaoQ indices were highest in fixed sand dunes and lowest in semi-fixed sand dunes.

### 3.2. Characterizing Variations in Functional Traits of Plant Communities at Different Stages of Fixation in Sand Dunes

#### 3.2.1. Leaf Functional Traits

**Figure 2:**
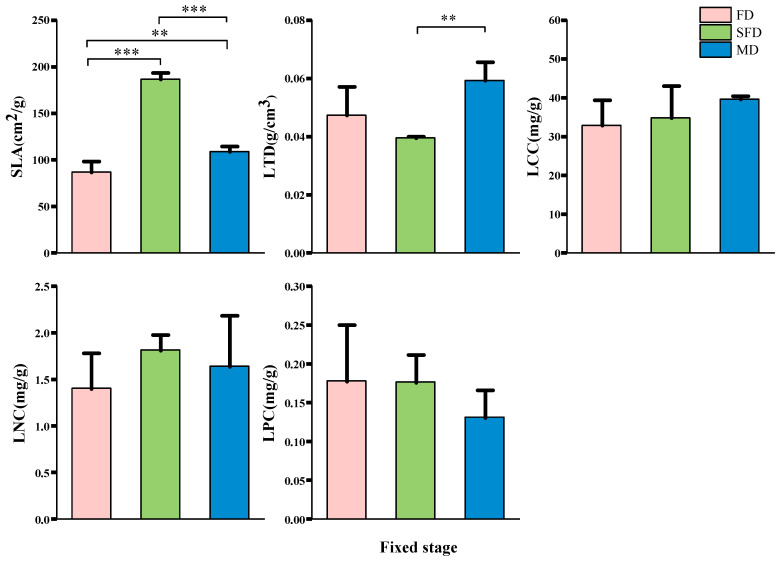
Differences in leaf functional traits of plant communities at different stages of fixation in sand dunes. Error bars indicate the standard error of the mean. Asterisks indicate significant differences (** *p* < 0.01, and *** *p* < 0.001). FD: Fixed dune; SFD: Semi-fixed dune; MD: Mobile dune; SLA: Specific leaf area; LTD: Leaf tissue density; LCC: Leaf carbon content; LNC: Leaf nitrogen content; LPC: Leaf phosphorus content.

Plant leaf functional traits varied significantly across dune fixation stages (Figure 2), with leaf morphological traits displaying greater variability than chemical traits. Semi-fixed dunes had an extremely significantly higher specific leaf area than both fixed and mobile dunes (*p* < 0.001), and mobile dunes showed a significantly higher specific leaf area than fixed dunes (*p* < 0.01). Additionally, leaf tissue density showed higher significance in mobile dunes compared to semi-fixed dunes (*p* < 0.01). No significant differences were observed in leaf carbon content (LCC), leaf nitrogen content (LNC), and leaf phosphorus content (LPC) across the different stages of dune fixation.

According to the “leaf economic spectrum,” the high specific leaf area and low leaf tissue density characteristics of semi-fixed and mobile sand dune plant communities indicate that they tend to favor a “fast investment–high return” strategy, while the low specific leaf area and high leaf tissue density characteristics of fixed sand dune plant communities indicate that they tend to favor a “slow investment–low return” strategy.

#### 3.2.2. Fine Root Functional Traits

**Figure 3:**
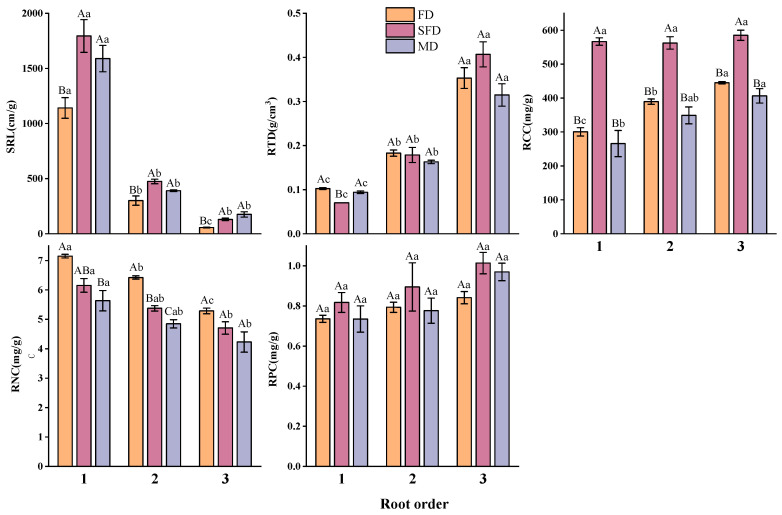
Differences in fine root functional traits of plant communities at different stages of fixation in sand dunes and different root orders. Different uppercase letters indicate significant differences in plant fine root traits among different stages of fixation in sand dunes (*p* < 0.05). Different lowercase letters indicate significant differences in plant fine root traits among root orders (*p* < 0.05). SRL: Specific root length; RTD: Root tissue density; RCC: Root carbon content; RNC: Root nitrogen content; RPC: Root phosphorus content. Error bars indicate the standard error of the mean.

The trends in plant fine root functional traits across different stages of dune fixation differed from those of leaf functional traits (Figure 3). Specific root length (SRL), root tissue density (RTD), root carbon content (RCC), and root nitrogen content (RNC) varied significantly across root orders (*p* < 0.05). SRL and RNC decreased with increasing root order, while RTD and RCC increased with increasing root order. The root phosphorus content (RPC) did not differ significantly (*p* > 0.05) at different fixation stages or among root orders. The differences in fine root functional traits across the different dune fixation stages were relatively small. SRL was significantly higher in the semi-fixed and mobile dune stages (*p* < 0.05) compared to the fixed dune stage. RCC was also significantly higher in the semi-fixed dune stage (*p* < 0.05) compared to both the fixed and mobile dune stages. RNC of primary and secondary root orders showed significant differences (*p* < 0.05) across different dune fixation stages, while tertiary root orders did not (*p* > 0.05). However, all root orders followed the trend: fixed dune > semi-fixed dune > mobile dune. RTD and RPC did not differ significantly across all root orders.

Similarly, according to the “root economic spectrum”, the high specific root length of semi-fixed and mobile sand dune plant communities indicates that they are more inclined toward a “fast investment–high return” strategy, while the specific root length of fixed sand dune plant communities indicates that they are more inclined toward a “slow investment–low return” strategy.

### 3.3. Correlations Between Plant Community Functional Traits and Plant Diversity

**Figure 4:**
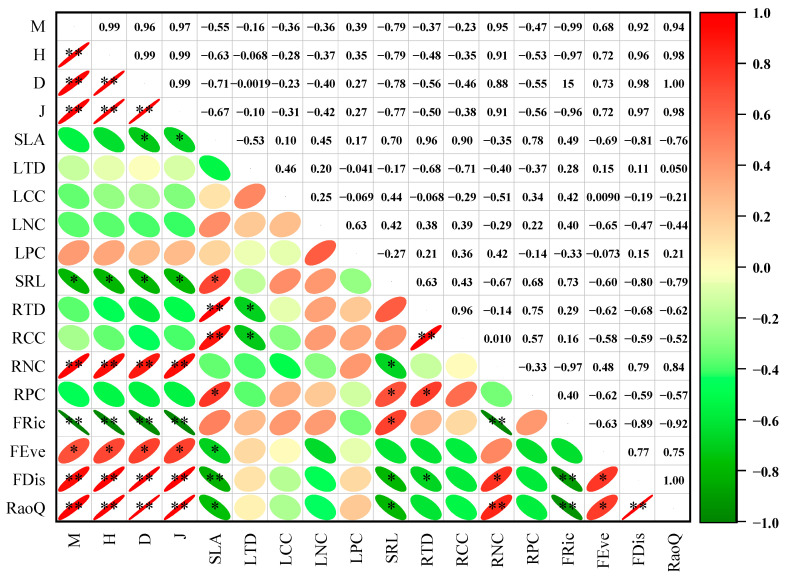
Pearson correlation analysis of plant community functional traits and plant diversity. Red and green encode positive and negative correlations, respectively. Asterisks indicate statistical significance: * *p* < 0.05, ** *p* < 0.01. M: Margalef index, H: Shannon–Wiener index, D: Simpson index, J: Pielou’s species evenness index; SLA: Specific leaf area; LTD: Leaf tissue density; LCC: Leaf carbon content; LNC: Leaf nitrogen content; LPC: Leaf phosphorus content; SRL: Specific root length; RTD: Root tissue density; RCC: Root carbon content; RNC: Root nitrogen content; RPC: Root phosphorus content; FRic: Functional richness; FEve: Functional evenness; FDis: Functional dispersion; RaoQ: Rao’s quadratic entropy.

Pearson’s correlation analysis (Figure 4) revealed a strong correlation between plant community functional traits and plant diversity in the transition zone of desert oases. Among related trait pairs, SLA and SRL exhibited a significant positive correlation, while STD and RTD showed a significant negative correlation. SLA, SRL, and RNC were strongly associated with both species diversity and functional diversity. Additionally, the Margalef index, Shannon–Wiener index, Simpson index, and Pielou’s evenness index showed highly significant positive correlations (*p* < 0.01) with the FDis and RaoQ indices, as well as a significant positive correlation with the FEve index (*p* < 0.05). A highly significant negative correlation was observed between these indices and the FRic index (*p* < 0.01). The results of the multiple stepwise regression analysis (Table 5) indicated that LTD, RNC, and SLA had the greatest influence on species and functional diversity. Specifically, LTD primarily impacted the Margalef index, RNC primarily affected species diversity, functional richness, functional discretization, and RaoQ, while SLA primarily influenced functional evenness.

### 3.4. Response of Plant Functional Traits and Community Composition to Environmental Changes

**Figure 5:**
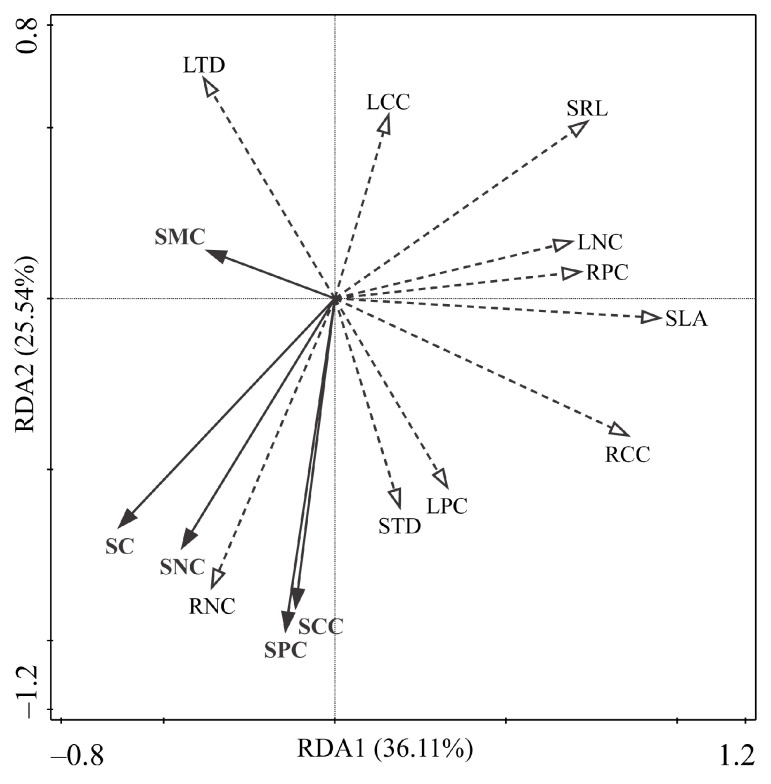
RDA ranking of plant functional traits and environmental factors. The solid and dashed arrows represent environmental factors and plant functional traits regulation, respectively. SLA: Specific leaf area; LTD: Leaf tissue density; LCC: Leaf carbon content; LNC: Leaf nitrogen content; LPC: Leaf phosphorus content; SRL: Specific root length; RTD: Root tissue density; RCC: Root carbon content; RNC: Root nitrogen content; RPC: Root phosphorus content; SC: Soil compaction; SMC: Soil moisture content; SCC: Soil carbon content; SNC: Soil nitrogen content; SPC: Soil phosphorus content.

The redundancy analysis (RDA) ordination plot (Figure 5) demonstrated that the first two axes, RDA1 and RDA2, explained 36.11% and 25.54% of the variance, respectively, with a cumulative explanation of 61.65%. This suggests that soil conditions play a significant role in the variation of functional traits across different vegetation zones. The influence of various soil factors on plant community functional traits is intricate. SC, SCC, SNC, and SPC exhibit positive correlations with RNC, STD, and LPC, while showing negative correlations with LCC and SRL; SMC is positively correlated with LTD and negatively correlated with SLA and RCC. Permutation tests of the explanatory variables (soil factors) within the RDA model (Table 6) revealed that SPC had a highly significant explanatory power concerning community traits, whereas SC exhibited a significant level of explanation. Specifically, SPC and SC accounted for 28.3% and 25.8% of the variance, respectively, indicating that soil phosphorus content is the key edaphic factor influencing variations in plant community functional traits across different dune fixation stages, followed by soil carbon content. Bio-Env analysis identified SPC as the most effective single environmental factor in explaining the observed variations in plant community composition across dune fixation stages, with a correlation coefficient of 0.785. The optimal model, combining SC and SCC, yielded a higher correlation coefficient of 0.896.

### 3.5. Analysis of Factors Influencing Plant Community Functional Diversity and Their Mechanisms

**Figure 6:**
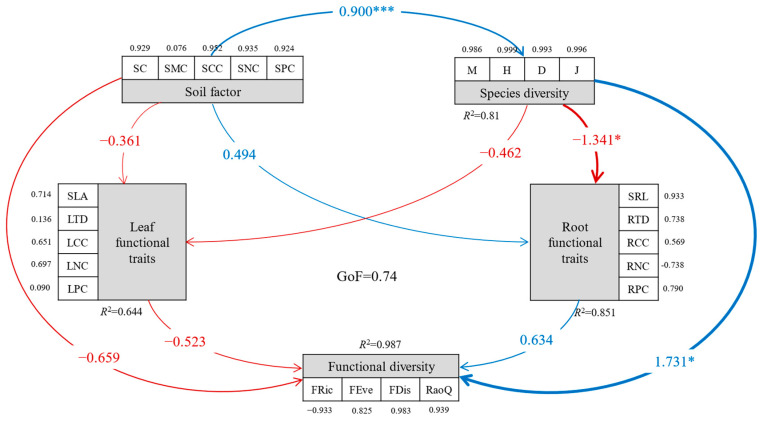
Direct and indirect effects of soil factors, species diversity, leaf and root functional traits on functional diversity. The blue and red arrows represent positive and negative regulation, respectively. The values on the arrows represent standardized path coefficients. The thickness of the arrows connecting the latent variables is proportional to the standardized path coefficients. The values near the arrows connecting the latent variables and the measured variables represent factor loadings. GoF represents goodness-of-fit. * *p* < 0.05, *** *p* < 0.001. *M*: Margalef index, *H*: Shannon–Wiener index, *D*: Simpson index, *J*: Pielou’s species evenness index; SLA: Specific leaf area; LTD: Leaf tissue density; LCC: Leaf carbon content; LNC: Leaf nitrogen content; LPC: Leaf phosphorus content; SRL: Specific root length; RTD: Root tissue density; RCC: Root carbon content; RNC: Root nitrogen content; RPC: Root phosphorus content; FRic: Functional richness; FEve: Functional evenness; FDis: Functional dispersion; RaoQ: Rao’s quadratic entropy; SC: Soil compaction; SMC: Soil moisture content; SCC: Soil carbon content; SNC: Soil nitrogen content; SPC: Soil phosphorus content.

Utilizing partial least squares structural equation modeling (PLS-SEM), we analyzed the effects of habitat changes on plant community functional diversity and identified potential underlying mechanisms. The model demonstrated a good fit, with a goodness-of-fit index of 0.74. The results (Figure 6) indicated that the model accounted for 98.7% of the variance in functional diversity. Species diversity had a direct positive effect on functional diversity and an indirect negative effect through fine root functional traits. Soil factors influenced functional diversity and fine root functional traits indirectly by significantly affecting species diversity. However, fine root and leaf functional traits did not have a significant direct impact on functional diversity. Additionally, the combined effect of soil factors and species diversity on functional diversity was substantial, with soil factors affecting functional diversity indirectly and species diversity exerting a direct positive effect.

## 4. Discussion

### 4.1. Habitat Differences-Induced Community Recombination Led to Significant Alterations in the Functional Traits of Plant Communities

Sand dune fixation leads to changes in environmental factors such as hydrothermal conditions and soil nutrients, resulting in environmental differences that further influence the structure, stability, and diversity of plant communities. The results are consistent with the hypothesis i, significant differences in community composition and diversity are observed in heterogeneous habitats resulting from varying degrees of dune fixation. Analysis of community composition variations and community-weighted mean (CWM) traits in these habitats reveals substantial variations in leaf and fine root traits across different fixation stages, accompanied by pronounced environmental gradient differences.

Studies have demonstrated that as sand dunes stabilize, vegetation coverage increases and has been accompanied by enhancements in species numbers and richness (Table 2, Figure 1). Many studies have found that in arid and semi-arid regions, both vegetation coverage and species richness increase as sand dunes stabilize, which are consistent with our research results [58,59]. Liu et al. [60] had observed that with the stability of sand dunes, species richness would first increase and then decrease or remained unchanged [61]. We found that the Margalef index, Shannon–Wiener index, Simpson index, and Pielou’s evenness index of fixed dune plant communities were consistently higher than those of other habitats, indicating stronger habitat stability. The increased availability of resources (e.g., water, nutrients) provides niche space for the coexistence of more species. In addition, the vegetation composition of fixed sand dunes is mainly composed of shrubs and perennial herbs. During the growing season, they reproduce asexually through organs such as stolons and rhizomes to allocate resources [62], rapidly completing growth and reproduction to restore the structure and diversity of plant communities. Conversely, the harsh conditions of mobile dunes (such as strong wind erosion and poor substrate) impose significant environmental filters, permitting only a few pioneer species to establish, resulting in a less diverse community structure.

Previous studies have demonstrated that both above-ground and below-ground plant traits respond to habitat changes [63]. Consequently, variations in leaf and fine root functional traits across different habitats reflect plants’ phenotypic plasticity in response to resource differences during dune stabilization (adaptive evolution). In our study, fixed dunes were predominantly vegetated by shrubs and perennial herbs, whereas semi-fixed and mobile dunes were primarily composed of annual herbs (Table 2 and Table 3). The higher specific leaf area and lower leaf dry matter content observed in semi-fixed dunes may result from differences in community composition driven by varying resource conditions. These conditions likely favor a “fast investment–high return” strategy, enhancing photosynthetic efficiency and facilitating rapid life cycle completion. In contrast, the dominant vegetation in fixed dunes—sandy shrubs and perennial herbs—exhibits lower specific leaf area and higher leaf dry matter content, indicative of a “slow investment–low return” strategy. This strategy is associated with longer leaf lifespans and reduces photosynthetic activity compared to annual herbs. Notably, although mobile dune vegetation shares a similar community composition with semi-fixed dunes, the harsher habitat conditions cause plant leaves to become thicker, and thicker leaves usually have thicker cell walls and mesophyll. In arid and infertile environments, plant leaves often develop thicker cell walls and mesophyll layers, investing more resources in leaf structure development, and resisting mechanical damage from wind and sand and water stress by thickening cell walls or cuticles. [64,65]. Thus, vegetation on mobile dunes has experienced continuous adaptive evolution, resulting in a reduced specific leaf area and an increased leaf dry matter content. These modifications promote the development of defensive structures that are crucial for survival in harsh environments.

The differentiation of functional traits among fine roots provides insight into the trade-off mechanisms governing subsurface resource allocation. Specifically, our study found that the specific root length across all root orders was significantly higher in semi-fixed and mobile dune stages compared to fixed dunes (Figure 3). This indicates that plant communities in semi-fixed and mobile dunes favor a rapid growth strategy through root elongation, enhancing nutrient uptake and adaptation to more challenging environments. Additionally, numerous studies have shown that root sequences exert a greater influence on ecosystem dynamics than tree species [66,67], and have a more pronounced effect on root functional traits than growth forms [47], implying that root sequences should be even less neglected in the study of root structure and functions. Our research further demonstrated that the impact of root order on root functional traits surpassed that of habitat. Specifically, SRL and root nitrogen content decreased, while root tissue density and carbon content increased with ascending root order, aligning with the observations of Beyer [66] and Yu et al. [68]. This pattern may arise because lower-order roots exhibit higher metabolic activity and primarily function in water and nutrient absorption, whereas higher-order roots are dedicated to storage. Consequently, the physiological processes within root systems are better characterized by a functional classification based on root order. This functional distinction supports the concept of the root economic spectrum (RES) [69].

### 4.2. Functional Traits and Diversity Synergize to Reflect Adaptive Strategies for Plant Community Survival

The relationships among plant functional traits reflect plants’ adaptations to habitat variations, illustrating their survival strategies [70]. Robust correlations between plant community functional traits and diversity further indicate species-environment interactions and species-specific constraints, which significantly influence species selection, trait optimization, community composition, and regulation [71]. Our study reveals strong correlations among species diversity, functional diversity, and functional traits within desert plant communities (Figure 4), which is consistent with the hypothesis ii.

The functional similarity hypothesis, as proposed by Chapin [34] and others, posits that pairs of similar traits in roots and leaves should exhibit comparable functions, resulting in strong correlations between them. In our study, we observed that similarity traits associated with resource acquisition in both roots and leaves were positively correlated with specific leaf area and specific root length. Conversely, traits linked to nutrient retention and defense mechanisms showed negative correlations with leaf and root tissue densities, aligning with the hypothesis and corroborating findings by Liu et al. [38]. Notably, the correlation between chemical traits in leaves and roots was weaker compared to that of morphological traits, consistent with the findings of Hobbie et al. [37]. Interestingly, three pairs of dissimilar traits—specific leaf area and root tissue density, specific leaf area and root carbon content, and root tissue density and root carbon content—demonstrated significant positive correlations. This suggests the existence of unique and flexible trait combinations between above-ground and below-ground plant species within the community, potentially as an adaptive response to varying environmental gradients [26].

The relationship between species diversity and functional diversity is fundamental to understanding the mechanisms underlying ecosystem functions. Extensive research indicates that increased species diversity can broaden the spectrum of functional traits within a community, thereby enhancing functional diversity [72,73]. Consequently, community functional characteristics are significantly influenced by species composition and distribution. However, some scholars argue that the mere presence of species does not necessarily translate into functional differences among them [74], and that the relationship between species diversity and functional diversity is not straightforward [75]. The eco-evolutionary community model proposed by Díaz [72] suggests that, over evolutionary timescales, species diversity may not always promote functional diversity and could even reduce it. Additionally, the relationship between species and functional diversity along environmental gradients can vary due to differing ecological processes [76]. The evolutionary processes involved in adapting to environmental gradients may hinder the positive monotonic relationship between species and functional diversity, potentially even reversing it [72].

A further examination using multivariate stepwise regression to assess how functional characteristics shape plant biodiversity demonstrated that the key drivers of both taxonomic richness and functional diversity are leaf tissue density, fine root nitrogen concentration, and specific leaf area (Table 5). Additionally, the carbon content in leaves and roots also contributed to these patterns. These effects may be attributed to the strong environmental filtering in arid regions, where plant communities must enhance their nutrient acquisition and utilization efficiency to cope with abiotic stresses such as nutrient deprivation and water scarcity. Consequently, leaf traits related to plant adaptation to habitat conditions (specific leaf area) and resource use efficiency (leaf tissue density) showed significant correlations with both species and functional diversity. Nitrogen, a key limiting factor for plant growth in arid zones [77], is particularly critical in desert ecosystems where soil nitrogen supports plant growth and is essential for assessing soil nutrient content [78]. Increased nitrogen content in fine roots may facilitate species coexistence by enhancing nitrogen uptake efficiency and reducing resource competition pressure. Furthermore, the negative correlation between functional richness and fine root nitrogen content may be due to plants with high nitrogen use efficiency in nutrient-poor habitats, which inhibit the establishment of species with low nitrogen use efficiency through ecological niche competition.

### 4.3. Soil Factors Regulate Fine Root Functional Traits and Diversity in Desert Communities by Influencing Species Diversity, Which in Turn Drives Community Succession

Desertification, driven by global climate change and increasing human activities, significantly contributes to soil nutrient loss and alteration of soil properties [78]. Along the desertification gradient, biomass (including above-ground, below-ground, and litter) declines markedly. Intense wind-induced soil erosion damages soil structure, leading to increased sandiness and decreased nutrient contents [79]. At the regional scale, soil factors are pivotal in shaping plant community characteristics. Environmental changes often result in shifts in species composition and community structure [75], with alterations in soil properties driving corresponding variations in plant communities and species diversity [80].

Our study employed redundancy and Bio-Env analyses to identify soil phosphorus content and soil compactness as the primary factors influencing variations in functional traits and community composition of desert plant communities, different from our initial hypothesis that soil moisture content would be the dominant factor (Figure 5, Table 6). Phosphorus is a critical element for ecosystem primary productivity and multifunctionality, essential for maintaining community productivity and stability [81]. In desert ecosystems, phosphorus availability is primarily determined by the weathering of parent rocks. The arid climate and low rainfall in these regions hinder phosphorus release by affecting rock weathering and exacerbating nutrient loss through intense wind erosion and sand movement. These factors collectively reduce phosphorus effectiveness in the soil, directly impacting the growth and reproduction of desert plants. Soil compactness drives resource capture trade-offs in plant communities. This is evidenced by the negative correlation between soil compactness and specific root length. Higher compactness, as seen in habitats like fixed dunes, limits fine root branching. Consequently, plant communities adopt a “slow investment–low return” strategy. This involves developing shorter, thicker root systems to minimize the costs of penetrating compacted soil. More interestingly, soil moisture content increased with the process of desertification, while mobile dunes had higher soil moisture content, likely due to increased transpiration associated with enhanced vegetation cover during dune fixation, leading to a gradual decrease in soil moisture content [82]. This observation potentially accounts for the limited impact of soil moisture on variations in both functional traits and community composition within desert plant communities, as observed in our study.

Recent studies have highlighted functional diversity as a crucial parameter for characterizing community structure and functional attributes in exploring community developmental succession, particularly in understanding community construction and ecosystem functions [83]. Variations in the functional characteristics of plant communities are influenced not only by community functional traits and species diversity, but also by soil nutrients and other site-specific conditions [84]. At the same time, Soil physicochemical properties can affect the distribution patterns of functional community traits by altering species diversity, thereby influencing the functional trait composition. While many studies have explored the interrelationships among environmental factors, plant diversity, and functional traits [85,86], the causal relationships and their impacts on community functional traits remain unclear. Our study found that soil factors indirectly influenced the functional diversity of plant communities by affecting species diversity. Notably, variations in species diversity did not directly affect functional diversity, although they negatively impacted root functional traits.

Therefore, based on our study’s findings and the “soil—diversity—community function trait” linkage mechanisms, we recommend prioritizing soil enhancement in the ecological restoration of desert sandy regions. In mobile dune areas, mitigating soil phosphorus limitations can be achieved by applying phosphorus fertilizers and incorporating organic matter. This approach not only promotes the formation of soil aggregates through organic matter decomposition but also improves soil compactness, enhancing its capacity to retain water and nutrients.

## 5. Conclusions

**Figure 7:**
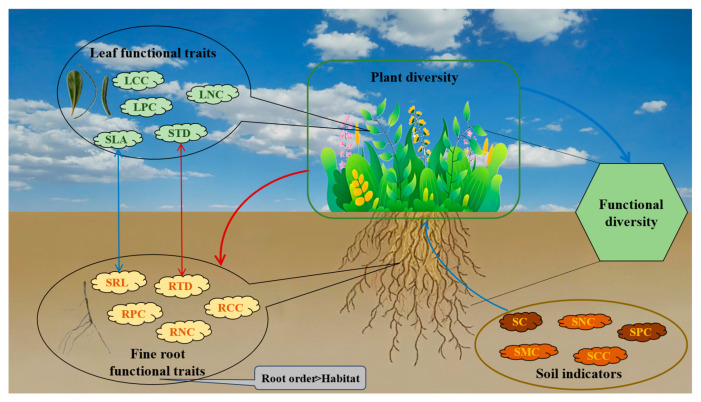
Summary model of direct and indirect relationships between plant attributes (plant diversity and plant traits) and soil indicators. The blue and red lines indicate positive and negative relationships, respectively. The thickness of the arrows connecting the latent variables is proportional to the standardized path coefficients. The pairs of functional traits connected by bidirectional arrows indicate significant correlations. Deeper colored soil indicators indicate stronger effects on plant community functional traits and community composition. SLA: Specific leaf area; LTD: Leaf tissue density; LCC: Leaf carbon content; LNC: Leaf nitrogen content; LPC: Leaf phosphorus content; SRL: Specific root length; RTD: Root tissue density; RCC: Root carbon content; RNC: Root nitrogen content; RPC: Root phosphorus content.

Overall, our study found that there were significant differences in the structure, diversity, and functional traits of plant communities in habitats with different degrees of desertification at different stages of sand dune fixation. Along the habitat gradient from fixed dunes to semi-fixed dunes and mobile dunes, plant community structure became increasingly homogeneous. Responses to abiotic stresses shifted from pressure-avoidance strategies to rapid resource acquisition strategies. A strong coupling was observed between functional traits and plant diversity. Based on these findings, we developed a model illustrating the relationships between plant attributes and soil factors (Figure 7). Desert plant communities adapted to habitat changes by coordinating above-ground and below-ground traits. Among pairs of similar traits, specific leaf area significantly correlated with specific root length, and leaf tissue density significantly correlated with root tissue density. For fine root functional traits, root order was more significantly affected than habitat for root functional traits. Soil phosphorus content and compactness were key factors affecting functional traits and community composition. Soil factors indirectly influenced functional diversity and fine-root traits by mediating species diversity. Thus, the discovery of this linkage-driven mechanism will be a key factor in predicting community succession and restoring ecosystem functions in desert sandy areas toward the “Holy Grail”.

## Figures and Tables

**Table 1:** Basic situation of soil at different stages of sand dune fixation.

Soil Indicators	Fixed Dune	Semi-Fixed Dune	Mobile Dune
SC (Pa)	35.555 ± 1.647	17.720 ± 0.903	15.952 ± 2.582
SMC (%)	1.375 ± 0.460	2.292 ± 0.591	4.012 ± 0.930
SCC (mg/g)	1.151 ± 0.015	0.993 ± 0.017	0.757 ± 0.018
SNC (mg/g)	0.091 ± 0.007	0.058 ± 0.003	0.046 ± 0.008
SPC (mg/g)	0.095 ± 0.002	0.074 ± 0.004	0.043 ± 0.004

**Table 2:** Main plant information of plant community in ecotone between desert and oasis.

Number	Species	Family	Genus	Life Forms
1	*Calligonum arborescens*	*Polygonaceae*	*Calligonum*	Shrub
2	*Haloxylon ammodendron*	*Chenopodiaceae*	*Haloxylon*	Shrub
3	*Nitraria sphaerocarpa*	*Zygophyllaceae*	*Nitraria*	Shrub
4	*Caragana korshinskii*	*Leguminosae*	*Caragana*	Shrub
5	*Reaumuria songarica*	*Tamaricaceae*	*Reaumuria*	Shrub
6	*Karelinia caspia*	*Compositae*	*Karelinia*	Perennial herb
7	*Cynanchum chinense*	*Asclepiadaceae*	*Cynanchum*	Perennial herb
8	*Salsola passerina*	*Chenopodiaceae*	*Salsola*	Perennial herb
9	*Inula salsoloides*	*Compositae*	*Inula*	Perennial herb
10	*Artemisia desertorum*	*Compositae*	*Artemisia*	Perennial herb
11	*Echinops gmelini*	*Compositae*	*Echinops*	Annual herb
12	*Suaeda glauca*	*Chenopodiaceae*	*Suaeda*	Annual herb
13	*Bassia dasyphylla*	*Chenopodiaceae*	*Bassia*	Annual herb
14	*Halogeton arachnoideus*	*Chenopodiaceae*	*Halogeton*	Annual herb
15	*Agriophyllum squarrosum*	*Chenopodiaceae*	*Agriophyllum*	Annual herb

**Table 3:** Vegetation characteristics and species relative abundance at different stages of fixation in sand dunes.

Fixed Stage	Dominant Species	Common Species
Fixed dune	*Karelinia caspia* (26.83%) *Reaumuria songarica* (14.48%) *Salsola passerina* (7.56%) *Echinops gmelini* (7.05%) *Bassia dasyphylla* (7.05%) *Nitraria sphaerocarpa* (6.30%) *Artemisia desertorum* (5.79%)	*Cynanchum chinense* (4.79%) *Inula salsoloides* (4.03%) *Halogeton arachnoideus* (3.90%) *Suaeda glauca* (3.65%) *Haloxylon ammodendron* (3.15%) *Caragana korshinskii* (2.77%) *Calligonum arborescens* (2.64%)
Semi-fixed dune	*Bassia dasyphylla* (81.08%) *Agriophyllum squarrosum* (13.07%)	*Nitraria sphaerocarpa* (2.89%) *Calligonum arborescens* (1.51%) *Haloxylon ammodendron* (1.45%)
Mobile dune	*Agriophyllum squarrosum* (76.00%) *Artemisia desertorum* (20.09%)	*Nitraria sphaerocarpa* (3.91%)

**Table 4:** Functional diversity of plant communities at different stages of fixation in sand dunes.

Functional Diversity	Fixed Dune	Semi-Fixed Dune	MOBILE DUNE
FRic	0.010 ± 0.008 c	6.621 ± 0.192 b	8.514 ± 0.045 a
FEve	0.811 ± 0.024 a	0.414 ± 0.029 b	0.579 ± 0.149 ab
FDis	2.887 ± 0.044 a	1.148 ± 0.082 c	1.601 ± 0.033 b
RaoQ	8.718 ± 0.294 a	2.620 ± 0.264 c	3.743 ± 0.120 b

FRic: Functional richness; FEve: Functional evenness; FDis: Functional dispersion; RaoQ: Rao’s quadratic entropy. Different lowercase letters indicate significant differences in functional diversity of plant communities among different stages of fixation in sand dunes (*p* < 0.05).

**Table 5:** Multivariate stepwise analysis between plant community functional traits and plant diversity.

Stepwise Regression Equation	*R* ^2^	*F*	Significant
*M* = −8.359 + 1.496 RNC + 22.471 LTD	0.939	62.706	*p* < 0.001
*H* = −3.605 + 1.031 RNC − 0.002 RCC	0.955	85.697	*p* < 0.001
*D* = −1.216 + 0.349 RNC − 0.001 RCC + 0.05 LCC	0.990	255.519	*p* < 0.001
*J* = −0.750 + 0.210 RNC − 0.001 RCC	0.976	163.659	*p* < 0.001
FRic = 36.474 − 6.145 RNC + 0.006 RCC	0.968	120.947	*p* < 0.001
FEve = 1.018 − 0.003 SLA	0.400	6.327	*p* < 0.05
FDis = −3.932 − 0.010 SLA + 0.902 RNC + 0.031 LCC	0.987	206.769	*p* < 0.001
RaoQ = −10.542 + 3.888 RNC − 0.014 RCC	0.976	160.516	*p* < 0.001

**Table 6:** Redundancy analysis replacement test for different environmental factors.

Parameters	Explains/%	Contribution/%	*F*	*p*
SC	**25.8**	**36.4**	**2.4**	**0.034**
SMC	4.0	5.7	0.4	0.724
SCC	6.0	8.5	0.8	0.616
SNC	6.9	9.6	0.8	0.54
SPC	**28.3**	**39.8**	**3.7**	**0.008**

SC: Soil compaction; SMC: Soil moisture content; SCC: Soil carbon content; SNC: Soil nitrogen content; SPC: Soil phosphorus content. The bold values denote significant terms at *p* < 0.05.

## Data Availability

The basic data were sourced from Chen et al. (2024) [47], and the functional trait values of plant communities were calculated in this study based on these data. Other data generated or analyzed during this study are included in this published article.
